# Gender Differences in Emergency Medicine Attending Physician Comments to Residents: A Qualitative Analysis

**DOI:** 10.1001/jamanetworkopen.2022.43134

**Published:** 2022-11-21

**Authors:** Mira Mamtani, Frances Shofer, Kevin Scott, Dana Kaminstein, Whitney Eriksen, Michael Takacs, Andrew K. Hall, Anna Weiss, Lauren A. Walter, Fiona Gallahue, Lainie Yarris, Stephanie B. Abbuhl, Jaya Aysola

**Affiliations:** 1 Department of Emergency Medicine, Penn Medicine, Philadelphia, Pennsylvania; 2 FOCUS on Health and Leadership for Women, Penn Medicine, Philadelphia, Pennsylvania; 3 Director of Epidemiology and Biostatistics, Department of Emergency Medicine, Penn Medicine, Philadelphia, Pennsylvania; 4 Co-Director of the Educational Research Program, Penn Graduate School of Education, Philadelphia, Pennsylvania; 5 Masters in Medical Education Program, Penn Graduate School of Education, Philadelphia, Pennsylvania; 6 Mixed Methods Research Lab, Penn Medicine, Philadelphia, Pennsylvania; 7 Department of Emergency Medicine, University of Iowa Carver College of Medicine, Iowa City; 8 Department of Emergency Medicine, University of Ottawa, Ottawa, Ontario, Canada; 9 Royal College of Physicians and Surgeons of Canada, Ottawa, Ontario, Canada; 10 Department of Pediatrics, Penn Medicine, Philadelphia, Pennsylvania; 11 Division of Emergency Medicine, Children’s Hospital of Philadelphia, Philadelphia, Pennsylvania; 12 Department of Emergency Medicine, University of Alabama at Birmingham, Birmingham; 13 Department of Emergency Medicine, University of Washington, Seattle; 14 Department of Emergency Medicine, Oregon Health and Sciences University, Portland; 15 Division of General Internal Medicine, Department of Internal Medicine, Penn Medicine, Philadelphia, Pennsylvania; 16 Penn Medicine Center for Health Equity Advancement, Penn Medicine, Philadelphia, Pennsylvania

## Abstract

**Importance:**

Prior studies have revealed gender differences in the milestone and clinical competency committee assessment of emergency medicine (EM) residents.

**Objective:**

To explore gender disparities and the reasons for such disparities in the narrative comments from EM attending physicians to EM residents.

**Design, Setting, and Participants:**

This multicenter qualitative analysis examined 10 488 narrative comments among EM faculty and EM residents between 2015 to 2018 in 5 EM training programs in the US. Data were analyzed from 2019 to 2021.

**Main Outcomes and Measures:**

Differences in narrative comments by gender and study site. Qualitative analysis included deidentification and iterative coding of the data set using an axial coding approach, with double coding of 20% of the comments at random to assess intercoder reliability (κ, 0.84). The authors reviewed the unmasked coded data set to identify emerging themes. Summary statistics were calculated for the number of narrative comments and their coded themes by gender and study site. χ^2^ tests were used to determine differences in the proportion of narrative comments by gender of faculty and resident.

**Results:**

In this study of 283 EM residents, of whom 113 (40%) identified as women, and 277 EM attending physicians, of whom 95 (34%) identified as women, there were notable gender differences in the content of the narrative comments from faculty to residents. Men faculty, compared with women faculty, were more likely to provide either nonspecific comments (115 of 182 [63.2%] vs 40 of 95 [42.1%]), or no comments (3387 of 10 496 [32.3%] vs 1169 of 4548 [25.7%]; *P* < .001) to men and women residents. Compared with men residents, more women residents were told that they were performing below level by men and women faculty (36 of 113 [31.9%] vs 43 of 170 [25.3%]), with the most common theme including lack of confidence with procedural skills.

**Conclusions and Relevance:**

In this qualitative study of narrative comments provided by EM attending physicians to residents, multiple modifiable contributors to gender disparities in assessment were identified, including the presence, content, and specificity of comments. Among women residents, procedural competency was associated with being conflated with procedural confidence. These findings can inform interventions to improve parity in assessment across graduate medical education.

## Key Points

**Question:**

Is the written performance feedback emergency medicine (EM) residents receive associated with gender?

**Findings:**

In this qualitative study of narrative comments by EM faculty across 5 sites, significant differences were found in the presence, content, and specificity of resident performance feedback by gender. For example, women residents were more likely to be rated below level, with conflation of procedural competency with procedural confidence.

**Meaning:**

These findings of gender disparities in written performance feedback suggest opportunities to improve parity in assessment and advancement of women in graduate medical education.

## Teaser

This qualitative study explores gender disparities and the reasons for such disparities in the narrative comments from emergency medicine attending physicians to emergency medicine residents at 5 sites across the US.

## Introduction

Feedback that is timely, specific, and actionable is key in creating an effective learning environment for resident physicians.^1,2^ Effective feedback improves learner motivation, engagement, and perceived support, lower rates of burnout, and clinical outcomes.^3,4^ However, delivering effective and unbiased feedback remains challenging with studies indicating that it is difficult to divorce gender and gendered expectations during the feedback process.^5,6^

Recent studies reveal gender disparities within the assessment and feedback process across specialties, including family medicine,^7^ general surgery,^8^ internal medicine,^9^ and particularly notable within emergency medicine (EM) training programs. In 2013, a study across 8 EM training programs of competency-based assessments revealed that the rate of milestone attainment throughout training was higher for men as compared with women residents across all 23 EM sub-competencies.^10^ A follow-up single-site study of written feedback to postgraduate year (PGY) 3 residents revealed that, women compared with men, received inconsistent feedback when struggling.^11^ A national data set of EM resident milestone assessments by clinical competency committees (CCC) revealed that there were gender disparities in 4 of the 22 subcompetencies (ie, emergency stabilization, general approach to procedures, airway management, and vascular access) at the time of graduation.^12^ Gender bias has shown to affect women’s career selection, advancement, and leadership opportunities^13^; within the field of emergency medicine, gender parity has not yet been achieved, with women representing only 35.9% of EM residents, 28.3% of EM faculty, and 11% of EM department chairs.^14,15,16^

While these prior studies revealed gender bias in assessment and feedback within EM training programs, specifically revealing that men achieve competency ratings faster than women, none examined what factors contribute to this disparity. Understanding modifiable contributors to gender inequities in the feedback and assessment process is integral to improving the learning environment for all trainees. Within the conceptual framework of sociocultural learning, the educational paradigm that suggests learning happens in and is informed by its context, gender inequity and potential biases in the clinical and nonclinical learning environment could affect how trainees learn and perform.^17^ Furthermore, addressing potential inequities in the assessment process within the learning environment would directly improve how men and women trainees learn.^17^ This is consistent with national policy guidelines from both the Accreditation Council of Graduate Medical Education (ACGME) and the Association of American Medical Colleges (AAMC), prioritizing the creation of a learning environment that acknowledges and addresses biases.^18,19^ In this qualitative study, we sought to explore gender disparities, the reasons for such disparities, and the association of faculty gender with the narrative comments provided to residents, derived from an online assessment system, from 5 training programs over 3 years to inform future interventions to mitigate this disparity.

## Methods

The institutional review board at each of the 5 study sites (University of Pennsylvania, University of Washington, Oregon Health and Sciences University, University of Iowa, and University of Alabama) deemed the study exempt, and informed consent was waived because participant information was deidentified. We followed the Standards for Reporting Qualitative Research (SRQR) reporting guideline^27^ and the Sex and Gender Equity Research Guidelines.^28^

### Study Objectives

We conducted a qualitative analysis of narrative comments on EM resident assessment forms by EM attending physicians using an online assessment system (Medhub) during a 3-year period (July 1, 2015 to June 30, 2018) across 5 EM training programs: University of Pennsylvania, University of Washington, Oregon Health and Sciences University, University of Iowa, and University of Alabama. These narrative assessment comments were used as the primary source of written feedback to EM trainees during the study period. The study sites did not mandate implicit bias training for the attending physicians during the study period.

Our study examined gender differences in the (1) total number of narrative comments by study site, and study year; (2) thematic content of the narrative comments; (3) specificity of feedback; and (4) faculty perception of resident performance as compared with their peers. Because prior studies have incompletely characterized why EM women residents lag behind their male colleagues in attaining EM milestones, we examined a subset of narrative comments among residents who were assessed as performing below level to understand differences by gender.

### Study Participants and Setting

Our study population included all EM faculty and trainees at the study sites. Gender of the residents and attending physicians were self-identified and provided by the site investigators to the research team. No participants self-identified as nonbinary during the study period. Study sites were chosen to ensure a geographic diversity and adequate site participation within the online evaluation system. Study sites participated within the years 2015 to 2018.

### Data Analysis

#### Qualitative

The qualitative analysis for this study was performed from 2019 to 2021 by the Mixed Methods Research Lab (MMRL) at University of Pennsylvania.^20^ The MMRL is composed of a team of experts in qualitative and mixed methods research methods who use their specialized knowledge and skills to analyze and manage qualitative data sets. We extracted assessment data inclusive of 283 EM residents during the study period from Medhub at the 5 study sites. We categorized assessment data as no comment when only competency scoring was present and with comment if both competency scoring and narrative comments were present. After linking qualitative assessment data and resident gender data, the entire data set was deidentified for names and pronouns that may indicate gender and thereafter imported into NVivo^®^ for coding and analysis.^21^

#### Coding

The study authors provided the MMRL with the codebook by Mueller et al^11^ as a guide prior to coding. The MMRL used a multistage, multianalyst iterative process that included (1) refining the code book through an iterative review of the data set (eAppendix in the Supplement); (2) coding the de-identified data using an axial coding approach^22^; and (3) double coding by separate coder of 20% of the data set at random to assess intercoder reliability. In lieu of the more traditional qualitative study approach of analyzing data until thematic saturation has been achieved, we coded the entire data set to better characterize nuances in narrative comments to men and women residents.^10,23^ We also had co-codes related to (1) faculty assessment of resident performance as compared with their peers designated as at level, above level, or below level and (2) specificity of narrative comments, designated as nonspecific vs specific when there was feedback on a specific case or behavior. Notably, both men and women coders, who were not part of the study team, were employed by the MMRL to provide gender balance and avoid confirmatory bias in the coding process. The coders (W.E., A.S., and C.K.) were trained by the MMRL prior to initiation of coding. Because the coders were nonphysicians, EM attending physicians reviewed the coded data set with the coders to provide clinical context and ensure trustworthiness. All coding discrepancies were resolved by group consensus. Intercoder reliability using the κ coefficient^24^ revealed acceptable agreement among coders (κ, 0.84).

#### Thematic Analysis^25^

Data were unmasked to gender after coding. We used a postpositivist deductive approach when reviewing the unmasked coded data set to identify emerging themes.^26^ We examined (1) the gender differences in the percent of faculty that provided narrative comments within these codes, and (2) the gender differences in the percent of residents that received narrative comments within these codes. We highlighted gender differences that were greater than or equal to 5%, to delve into the areas with the greatest differences and reported these differences in summary form with identified emerging themes.

#### Descriptive Analysis of Qualitative Themes

Our qualitative data comprised 39 776 codes for all narrative comments during the study period. We calculated summary statistics (ie, frequencies and percentages) of the number of narrative comments and their themes by gender, study site, and overall. We used χ^2^ tests to identify differences in the proportion of narrative comments by gender in 4 separate dyads (ie, men faculty to men residents, men faculty to women residents, women faculty to men residents, and women faculty to women residents). Descriptive analyses were conducted using SAS statistical software version 9.4 (SAS Institute).^29^

## Results

### Gender Differences in Providing Narrative Comments

The study population included 283 EM residents, of whom 170 (60%) identified as men and 113 (40%) as women, and 277 EM attending physicians, of whom 182 (66%) identified as men and 95 (34%) women. Of the total 15 044 instances of assessment during the study period, 10 488 (69.7%) included both competency scorings and narrative comments (with comments) and 4556 (30.3%) included only competency scorings (no comments). Of the data with no comments, 3387 of 10 496 (32.3%) came from men faculty compared with 1169 of 4548 (25.7%) of women faculty (*P* < .001). Overall, women faculty provided more narrative comments to women residents compared with men residents (1814 of 2576 [79.4%] vs 1565 of 1972 [70.4%]; *P* < .001). Similarly, men faculty provided more comments to women vs men residents (2631 of 3713 [70.9%] vs 4478 of 6783 [66.0%]; *P* < .001).

**Figure 1.**
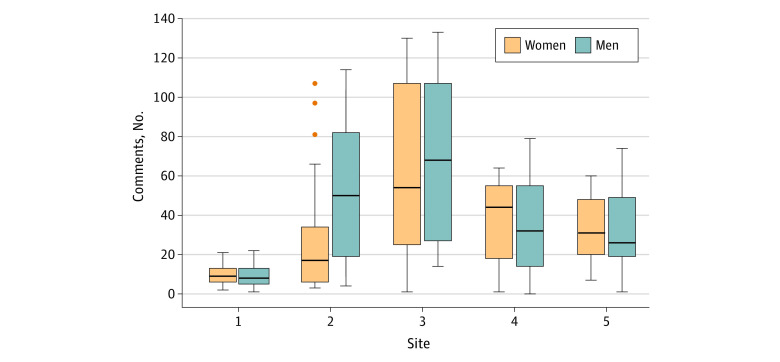
Distribution of Narrative Comments Received by Emergency Medicine Residents by Gender and Site Boxes indicate the IQR; dots, outliers; line within the box, median; error bars, 95% CI.

**Figure 2.**
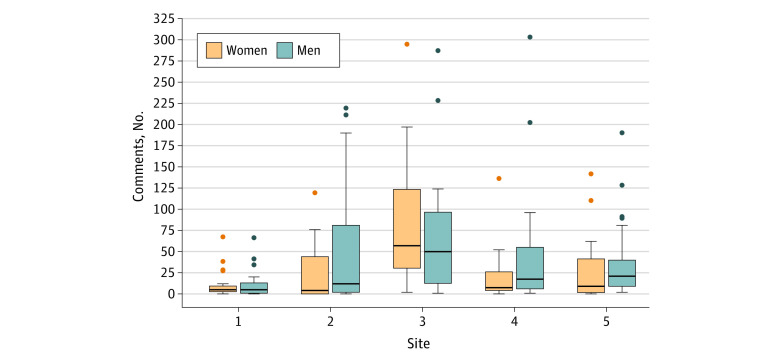
Distribution of Narrative Comments Provided by Emergency Medicine Faculty by Gender and Site Boxes indicate the IQR; dots, outliers; line within the box, median; error bars, 95% CI.

The gender distribution of residents and faculty as well as the number of narrative comments varied across study sites (Figure 1 and Figure 2). Site 1 had the fewest narrative comments (men and women faculty provided a median [IQR] of 5 [1-13] and 5 [2-10], respectively), while site 3 had the most narrative comments (men and women faculty provided a median [IQR] of 50 [13-97] and 57 [31-124], respectively). A greater proportion of attending physicians were men at each of the study sites. Within sites, we found no differences in the number of comments between men and women faculty or residents, except for site 2 which had more comments directed to men residents vs women residents (median [IQR], 50 [19-82] vs 17 [6-34]).

### Differences in the Content of Narrative Comments Provided by Men vs Women Faculty

**Figure 3.**
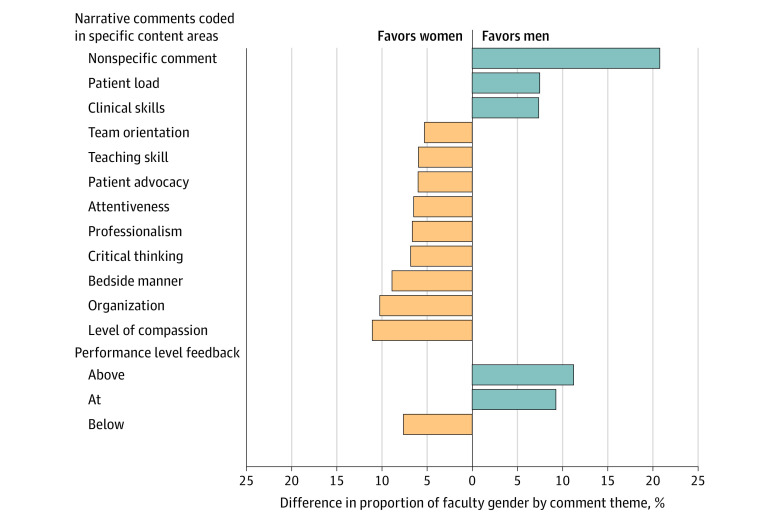
Comparisons of Feedback Themes Provided by Faculty Gender

Among the 10 488 instances in which narrative comment were provided, we found differences in coded feedback themes by faculty gender. Men faculty compared with women faculty provided more comments about patient load (100 of 182 [54.9%] vs 45 of 95 [47.4%]) and clinical skills (115 of 182 [63.2%] vs 53 of 95 [55.8%]). In contrast, women faculty compared with men faculty provided more comments about team orientation, teaching skills, patient advocacy, attentiveness, professionalism, critical thinking, bedside manner, organization and level of compassion, ranging from 60 of 95 (63.2%) vs 105 of 182 (57.7%) to 44 of 95 (46.3%) and 64 of 182 (35.2%). Figure 3 illustrates the proportion of faculty who provided feedback in specific content areas, highlighting only the instances in which there was greater than 5% difference between men and women faculty.

### Differences in the Content of Narrative Comments Received by Men vs Women Residents

**Figure 4.**
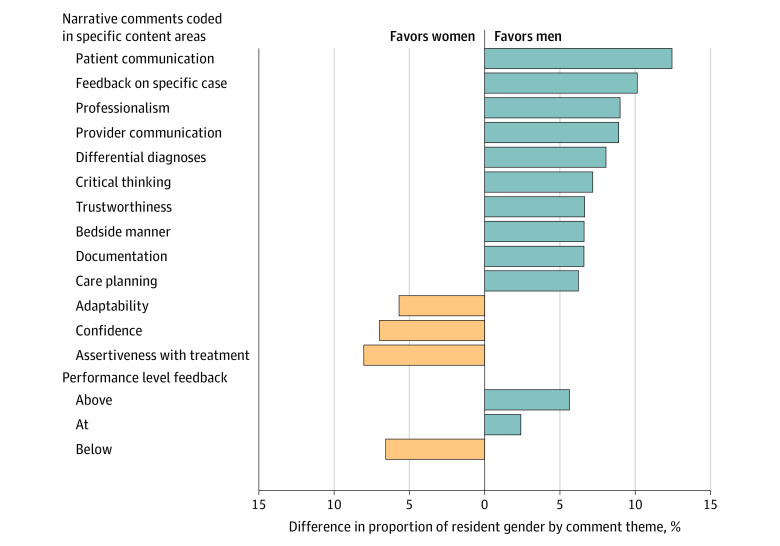
Comparisons of Feedback Themes Received by Resident Gender

More men trainees, compared with women trainees, received narrative comments about documentation, differential diagnosis, care planning, patient and clinician communication, critical thinking, bedside manner, trustworthiness, and professionalism, with differences ranging from 140 of 170 (82.4%) vs 86 of 113 (76.1%) to 131 of 170 (77.1%) vs 73 of 113 (64.6%). An example of a comment coded as communication skill included “they treat all patients with unconditional positive regard…and [brings] advanced communication skills to the team.” In contrast, more women residents as compared with men residents received narrative comments about confidence, assertiveness with treatment, and adaptability, with differences ranging from 31 of 113 (27.4%) vs 37 of 170 (21.8%) to 31 of 113 (27.4%) vs 33 of 170 (19.4%). An example of a comment coded as adaptability included: “resident is reliable but at times more independent than I prefer, and it makes me not always trust them—I haven’t seen them do harmful things, but I’ve had conversations with them where they dig their heels in, nearly refuses to act on my suggestion and takes a righteous stand.” Figure 4 illustrates the proportion of residents who received feedback in specific content areas, highlighting only the instances in which there is a greater than 5% difference between men and women residents.

### Differences in the Specificity of Narrative Comments Provided Between Men and Women Faculty

There were also differences in the specificity of comments provided by faculty gender. Men faculty provided feedback that was nonspecific more often than women faculty (115 of 182 [63.2%] vs 40 of 95 [42.1%]) (Figure 3). Examples of these nonspecific comments included: “Great work” and “Doing well, keep up the good work.” These nonspecific comments were directed equally to men and women residents and were consistent across study sites.

### Differences in the Specificity of Narrative Comments Received Between Men and Women Residents

Men and women residents also differed in the specificity of feedback received. Men residents received feedback on specific cases or behaviors more often than women residents (103 of 170 [60.6%] vs 57 of 113 [50.4%]) (Figure 4). Examples of men receiving feedback on specific cases included “Excellent management of patient with SVT unresponsive to conventional care. Good choice of meds and other management” and “Had a scare where they looked at the wrong dated CT on a patient which was negative when the most recent CT had a head bleed…we discussed the importance of right patient, right date, right time.” These patterns were consistent irrespective of faculty gender and across most of our study sites.

### Differences in Men vs Women Faculty Perception of Resident Performance

Among the 10 088 instances in which narrative comments were provided, 2556 instances (25.3%) included faculty commenting on resident performance. In this subset, more men faculty compared with women faculty assessed that the resident was performing above level (124 of 182 [68.1%] vs 54 of 95 [56.8%]) or at level (109 of 182 [59.9%] vs 48 of 95 [50.5%]) compared with their peers (Figure 3). In contrast, more women faculty compared with men faculty assessed that the resident was performing below level compared with their peers (28 of 95 [29.5%] vs 41 of 182 [22.5%]) (Figure 3).

### Differences in Men vs Women Resident Performance Feedback

More men residents were assessed as performing above level (compared with their peers (145 of 170 [85.3%] vs 90 of 113 [79.6%]), while more women residents were assessed as performing below level compared with their peers (36 of 113 [31.9%] vs 43 of 170 [25.3%]) (Figure 4). These patterns were consistent irrespective of faculty gender, and across most of our study sites.

### Below Level Comments to EM Trainees

Among the subset of residents with faculty assessment of below level performance, we observed that more than 5% of women trainees received comments that their procedural skills were below level compared with men (7 of 113 [6.19%] vs 2 of 170 [1.18%]). Furthermore, we identified that below-level procedural skill comments directed to women trainees were frequently co-coded with comments about confidence (or lack thereof). Examples included “Resident lacks some confidence also when it comes to procedures-given that they are at the end of their [postgrad year 2], they need to find more of them to build up their confidence” and “Resident appears to have solid clinical skills but has gotten flustered when procedures are required.”

In contrast, below-level procedural skill comments directed to men residents included specific actionable items without mention of their confidence with procedures. Examples included “But the last shift we worked together you missed a few things including…getting an opening pressure on a transplant patient in whom you were performing a lumbar puncture” and “Advised using direct laryngoscopy for very bloody airway.”

## Discussion

To our knowledge, this is the first multisite study to examine gender differences in narrative comments to EM residents. We analyzed 10 488 narrative comments submitted from 2015 to 2018 across 5 training programs and found gender differences in multiple domains, including whether narrative comments were provided to trainees, the content and specificity of the comments to trainees, and the perceptions of trainee performance. Among faculty, more men compared with women provided either no narrative comment feedback or provided nonspecific comments. Among trainees, women were more likely to receive narrative comments from both men and women faculty; however, those comments were less specific, and they were assessed as performing below level more often than men residents.

Prior studies have revealed that men achieve competency ratings faster than women residents. This study builds upon prior work to reveal what modifiable factors may be contributing to this disparity. Our qualitative analysis identified differences in perceived competency related to procedural skills by gender, with women trainees often noted to lack confidence with procedures, as a leading driver of the below level narrative feedback received by women trainees. This finding suggests that, among women residents, attending physician perception of resident confidence may be conflated with ratings of procedural competency. These findings, while identified in EM training programs, are translatable across graduate medical education.

Achieving mastery of procedural skills is an important component of clinical competency.^30^ Residents have a minimum number of procedures they need to complete in order to progress to independent practice.^31^ Prior studies have revealed no gender differences in procedural outcomes among EM physicians including first pass-intubation success^32^ and accuracy of cricothyroid membrane identification.^33^ However, both the Dayal et al^10^ and Santen et al^12^ studies revealed that among EM trainees, women residents lag behind men in achieving procedural subcompetency scores. Our study findings suggest that among women residents who are assessed as performing below level in procedural skills, competency with procedure may be conflated with confidence in executing the procedure. It is unclear whether women express less confidence in procedures or whether faculty perception of trainee confidence in procedures is leading to poorer ratings of procedural skills or overinflation of ratings among men. However, none of the comments included discussion of procedural outcomes.

Etiologies for this disparity are likely multifactorial but may be at least partially explained by gender and leadership norms in medicine. Historically, emergency medicine has roots in surgery and in the army, fields that have until recently been composed primarily of men with agentic or action-oriented and assertive leadership styles being lauded without accounting for the strengths of leadership styles that are not in line with this norm.^11,34,35^ Not surprisingly, among surgical training programs a similar phenomenon exists with women residents’ self-reporting lower autonomy in the operating room.^36^ In this context, it is possible that our collective definitions of clinical skills or procedural skills are so entrenched in a gendered model of competency that we do not have the appropriate tools to determine resident abilities that do not fit this model. One means to break out of this outdated mold would be to assess residents with more objective parameters. For example, a recent study^37^ identified that using checklist based procedural evaluations may mitigate gendered assessments. Alternatively, assessment of procedural competency could include reference to procedural outcomes, or clinical based competency could include discussion of patient outcomes or care patterns.^38^ It is notable that while our frameworks for assessment, such as the EM milestones,^39^ may not be inherently biased, the lens by which we interpret these models and apply them at the level of the CCC can lead to biased assessment. Given this reality, we may need to reconsider how we use these frameworks, particularly when using high-stakes advancement decisions.

Our study also suggests that the focus and volume of faculty feedback differs by resident gender. Women, compared with men, provide more narrative comments to residents. Providing feedback is integral to the growth and development of learners, and yet not recognized in the tenure and promotion process, consistent with prior literature that women engage in work that is valuable but often not rewarded by academic institutions.^40,41^ In addition, while women residents receive concentrated feedback on personal attributes, such as adaptability, assertiveness, and confidence, men residents receive feedback in multiple domains. This finding is consistent with prior studies across specialties indicating that women receive more comments about their personal attributes and disposition, compared with men who receive feedback in competency-related behaviors.^42,43^ Studies have shown that gender biases are prevalent, exist in both men and women,^44^ and that it is notoriously hard to train out biases. However, a recent study by Peterson et al^45^ examining student assessments of teachers revealed that identifying that biases exist prior to filling out assessment can mitigate the disparities seen. Training programs and institutions could consider including an opportunity for individuals engaging in assessment to understand and acknowledge their biases prior to providing narrative assessment comments.

Lastly, our data suggest that among faculty, men may be more likely to provide no comment or nonspecific comments, and among residents, men may be more likely to receive feedback on specific cases. While prior studies have revealed gender differences in the consistency of feedback to EM trainees,^11^ to our knowledge, this is first study to identify gender differences in specificity of feedback to EM residents. Nonspecific feedback may represent missed opportunities to assess skills and deficiencies.^46^ As women residents are more likely to be assessed as performing below level but less likely to receive feedback on specific cases or behaviors, compared with men, this may limit opportunities for growth and development throughout their training.^46^ These disparities can be propagated beyond residency training, given that assessments often inform letters of recommendations for employment or fellowships.^46^ As providing specific, actionable feedback is integral to resident growth and development,^2^ it is imperative that efforts are made to improve the specificity of feedback provided by EM faculty. Interventions such as real-time point of care nudges while completing narrative comments and faculty training sessions should be explored to address this critical need.

### Limitations

Our study had several limitations. While we identified gender differences in the narrative comments provided to EM trainees, demographic factors, such as age, race and ethnicity, sexual orientation, were not available in the data set, so we were unable to assess the multifactorial effects of these intersecting identities. In addition, as we had no participants who self-identified as nonbinary during the study period, we do not account for findings that may be relevant to those who identify on the gender continuum. Furthermore, while we have identified that our major findings are consistent across study sites, there may be additional site-specific variations influenced by local culture that should be explored in future studies. Lastly, as we chose to highlight gender differences that were greater than or equal to 5% in the narrative comments provided to trainees, we might have missed additional emerging themes that were notable in the data set for which the differences were less than 5%.

## Conclusions

In this qualitative study of narrative comments provided by EM faculty, we have identified multiple modifiable contributors that are associated with gender disparities in narrative comments to EM residents, including the presence, content, and specificity of the comments as well as identifying that among women residents procedural confidence may be conflated with procedural competency. The results of this study could inform future interventions to mitigate these disparities across graduate medical education.

## Notes

Supplement. **eAppendix.** Codebook With Description

## References

[1] Rodgers KG, Manifold C. 360-degree feedback: possibilities for assessment of the ACGME core competencies for emergency medicine residents. Acad Emerg Med. 2002;9(11):1300-1304. doi:10.1197/aemj.9.11.1300 12414485

[2] van de Ridder JM, Stokking KM, McGaghie WC, ten Cate OT. What is feedback in clinical education? Med Educ. 2008;42(2):189-197. doi:10.1111/j.1365-2923.2007.02973.x 18230092

[3] Frampton A, Fox F, Hollowood A, . Using real-time, anonymous staff feedback to improve staff experience and engagement. BMJ Qual Improv Rep. 2017;6(1):u220946.w7041. doi:10.1136/bmjquality.u220946.w7041 28469897 PMC5411713

[4] Sexton JB, Adair KC, Leonard MW, . Providing feedback following Leadership WalkRounds is associated with better patient safety culture, higher employee engagement and lower burnout. BMJ Qual Saf. 2018;27(4):261-270. doi:10.1136/bmjqs-2016-006399 28993441 PMC5867443

[5] Wenneras C, Wold A. Nepotism and sexism in peer-review. Nature. 1997;387(6631):341-343. doi:10.1038/387341a0 9163412

[6] Steinpreis REAK, Ritzke D. The impact of gender on the review of the curricula vitae of job applicants and tenure candidates: a national empirical study. Sex Roles. 1994;41(7):509-528.

[7] Loeppky C, Babenko O, Ross S. Examining gender bias in the feedback shared with family medicine residents. Educ Prim Care. 2017;28(6):319-324. doi:10.1080/14739879.2017.1362665 28812957

[8] Roshan A, Farooq A, Acai A, . The effect of gender dyads on the quality of narrative assessments of general surgery trainees. Am J Surg. Published online December 3, 2021. doi:10.1016/j.amjsurg.2021.12.001 34911639

[9] Klein R, Ufere NN, Rao SR, ; Gender Equity in Medicine workgroup. Association of gender with learner assessment in graduate medical education. JAMA Netw Open. 2020;3(7):e2010888. doi:10.1001/jamanetworkopen.2020.10888 32672831 PMC7366188

[10] Dayal A, O’Connor DM, Qadri U, Arora VM. Comparison of male vs female resident milestone evaluations by faculty during emergency medicine residency training. JAMA Intern Med. 2017;177(5):651-657. doi:10.1001/jamainternmed.2016.9616 28264090 PMC5818781

[11] Mueller AS, Jenkins TM, Osborne M, Dayal A, O’Connor DM, Arora VM. Gender differences in attending physicians’ feedback to residents: a qualitative analysis. J Grad Med Educ. 2017;9(5):577-585. doi:10.4300/JGME-D-17-00126.1 29075375 PMC5646913

[12] Santen SA, Yamazaki K, Holmboe ES, Yarris LM, Hamstra SJ. Comparison of male and female resident milestone assessments during emergency medicine residency training: a national study. Acad Med. 2020;95(2):263-268. doi:10.1097/ACM.0000000000002988 31517688 PMC7004441

[13] Edmunds LD, Ovseiko PV, Shepperd S, . Why do women choose or reject careers in academic medicine: a narrative review of empirical evidence. Lancet. 2016;388(10062):2948-2958. doi:10.1016/S0140-6736(15)01091-0 27105721

[14] Active physicians by sex and speciality. Association of American Medical Colleges. Accessed May 4, 2021. https://www.aamc.org/data-reports/workforce/interactive-data/active-physicians-sex-and-specialty-2019

[15] Number of active residents, by type of medical school, GME speciality, and sex: 2019-2020 active residents. Association of American Medical Colleges. Accessed May 4, 2021. https://www.aamc.org/data-reports/students-residents/interactive-data/report-residents/2020/table-b3-number-active-residents-type-medical-school-gme-specialty-and-sex

[16] Department Chairs by department, sex, and race/ethnicity, 2020. Association of American Medical Colleges. Accessed July 3, 2021. https://www.aamc.org/media/9066/download

[17] Shabani K. Applications of Vygotsky’s sociocultural approach for teacher’s professional development. Cogent Education. 2016;3(1):1-10. doi:10.1080/2331186X.2016.1252177

[18] CLER Pathways to Excellence: Expectations for an optimal clinical learning environment to achieve safe and high quality patient care. Accreditation Council for Graduate Medical Education. Accessed November 2, 2022. https://www.acgme.org/Portals/0/PDFs/CLER/CLER_Brochure.pdf

[19] Proceedings of the diversity and inclusion innovation forum: unconscious bias in academic medicine. Association of American Medical Colleges. Accessed November 2, 2022. https://store.aamc.org/downloadable/download/sample/sample_id/168/

[20] Mixed Methods Research. Perelmam School of Medicine. Accessed May 4, 2021. https://www.med.upenn.edu/fmch/mixed-methods-research-lab

[21] NVivo Qualitative Data Analysis Software. Version 11. QSR International Pty Ltd; 2015.

[22] Allen M. *The SAGE Encyclopedia of Communication Research Methods*. Vol 4. SAGE Publications, Inc; 2017. doi:10.4135/9781483381411

[23] Hanson JL, Balmer DF, Giardino AP. Qualitative research methods for medical educators. Acad Pediatr. 2011;11(5):375-386. doi:10.1016/j.acap.2011.05.001 21783450

[24] Viera AJ, Garrett JM. Understanding interobserver agreement: the kappa statistic. Fam Med. 2005;37(5):360-363. 15883903

[25] Guest G, MacQueen KM, Namey EE. Applied Thematic Analysis. *SAGE* Publications; 2012. Accessed November 2, 2022. https://methods.sagepub.com/book/applied-thematic-analysis

[26] Hess-Biber SN. The Practice of Qualitative Research: Engaging Students in the Qualitative Process. Boston College; 2016.

[27] O’Brien BC, Harris IB, Beckman TJ, Reed DA, Cook DA. Standards for reporting qualitative research: a synthesis of recommendations. Acad Med. 2014;89(9):1245-1251. doi:10.1097/ACM.0000000000000388 24979285

[28] Heidari S, Babor TF, De Castro P, Tort S, Curno M. Sex and gender equity in research: rationale for the SAGER guidelines and recommended use. Res Integr Peer Rev. 2016;1:2. doi:10.1186/s41073-016-0007-6 29451543 PMC5793986

[29] SAS. 9.4 Statements: Reference. SAS Institute Inc; 2013.

[30] Tran V, Cobbett J, Brichko L. Procedural competency in emergency medicine training. Emerg Med Australas. 2018;30(1):103-106. doi:10.1111/1742-6723.12925 29341458

[31] Emergency medicine defined key index procedure minimums. Accreditation Council for Graduate Medical Education. Accessed December 2, 2021. https://www.acgme.org/globalassets/pfassets/programresources/em_key_index_procedure_minimums_103117.pdf

[32] Jung W, Kim J. Factors associated with first-pass success of emergency endotracheal intubation. Am J Emerg Med. 2020;38(1):109-113. doi:10.1016/j.ajem.2019.09.001 31843066

[33] Alshareef H, Al Saawi A, Almazroua F, Alyami H, Reilly GO, Mitra B. Localisation of the cricothyroid membrane by digital palpation in the emergency department. Postgrad Med J. 2018;94(1114):442-445. doi:10.1136/postgradmedj-2018-135828 30126930

[34] Combat medic demogrpahic and statistics in the US. Zippia. Accessed August 20, 2022. https://www.zippia.com/combat-medic-jobs/demographics/?src=sp-popout-linkclick

[35] Daniels CM, Dworak TC, Anderson AB, . Gender disparities within US army orthopedic surgery: a preliminary report. Mil Med. 2018;183(1-2):e162-e166. doi:10.1093/milmed/usx061 29401339

[36] Cookenmaster C, Shebrain S, Vos D, Munene G, Miller L, Sawyer R. Gender perception bias of operative autonomy evaluations among residents and faculty in general surgery training. Am J Surg. 2021;221(3):515-520. doi:10.1016/j.amjsurg.2020.11.016 33189312

[37] See A, Pallaci M, Aluisio AR, . Assessment of implicit gender bias during evaluation of procedural competency among emergency medicine residents. JAMA Netw Open. 2022;5(2):e2147351. doi:10.1001/jamanetworkopen.2021.47351 35129594 PMC8822382

[38] Schumacher DJ, Martini A, Holmboe E, . Initial implementation of resident-sensitive quality measures in the pediatric emergency department: a wide range of performance. Acad Med. 2020;95(8):1248-1255. doi:10.1097/ACM.0000000000003147 31913878

[39] Emergency medicine milestones. Accreditation Council for Graduate Medical Education. Accessed September 3, 2021. https://www.acgme.org/Portals/0/PDFs/Milestones/EmergencyMedicineMilestones.pdf

[40] Murphy M, Callander JK, Dohan D, Grandis JR. Women’s experiences of promotion and tenure in academic medicine and potential implications for gender disparities in career advancement: a qualitative analysis. JAMA Netw Open. 2021;4(9):e2125843. doi:10.1001/jamanetworkopen.2021.25843 34542616 PMC8453318

[41] Greider CW, Sheltzer JM, Cantalupo NC, . Increasing gender diversity in the STEM research workforce. Science. 2019;366(6466):692-695. doi:10.1126/science.aaz0649 31699926

[42] Rojek AE, Khanna R, Yim JWL, . Differences in narrative language in evaluations of medical students by gender and under-represented minority status. J Gen Intern Med. 2019;34(5):684-691. doi:10.1007/s11606-019-04889-9 30993609 PMC6502922

[43] Gerull KM, Loe M, Seiler K, McAllister J, Salles A. Assessing gender bias in qualitative evaluations of surgical residents. Am J Surg. 2019;217(2):306-313. doi:10.1016/j.amjsurg.2018.09.029 30343879 PMC8687875

[44] Pham T. Think you’re not biased against women at work: read this. Forbes. Accessed October 18, 2022. https://www.forbes.com/sites/break-the-future/2016/12/20/think-youre-not-biased-against-women-at-work-read-this/?sh=15c31e57e5a8

[45] Peterson DAM, Biederman LA, Andersen D, Ditonto TM, Roe K. Mitigating gender bias in student evaluations of teaching. PLoS One. 2019;14(5):e0216241. doi:10.1371/journal.pone.0216241 31091292 PMC6519786

[46] Klein R, Julian KA, Snyder ED, ; From the Gender Equity in Medicine (GEM) workgroup. gender bias in resident assessment in graduate medical education: review of the literature. J Gen Intern Med. 2019;34(5):712-719. doi:10.1007/s11606-019-04884-0 30993611 PMC6502889

