# Supplementary material for: Gender Differences in Emergency Medicine Attending Physician Comments to Residents: A Qualitative Analysis
Source: JAMA Netw Open. 2022 Nov 21;5(11):e2243134. doi: 10.1001/jamanetworkopen.2022.43134 (PMC9679878; doi:10.1001/jamanetworkopen.2022.43134)
Supplement: Supplement. — eAppendix. Codebook With Description [file jamanetwopen-e2243134-s001.pdf]

Supplemental Online Content

Mamtani M, Shofer F, Scott K, et al. Gender differences in emergency medicine attending physician comments to residents: a qualitative analysis. *JAMA Netw Open*. 2022;5(11):e2243134. doi:10.1001/jamanetworkopen.2022.43134

eAppendix. Codebook With Description

This supplemental material has been provided by the authors to give readers additional information about their work.

© 2022 Mamtani M et al. *JAMA Network Open.*

eAppendix. Codebook with Description

Name	Description
**Clinical Practice**	
Adaptability	Flexibility to situation (how patient is presenting) and changes course if necessary, adaptability to treatment course, navigates well
Assertiveness with Treatment	Proactive in getting treatment for patient
Attentiveness	Present in the situation, focus, such as resuscitation/trauma event, or lack of attention
Bed Side Manner	focus on patient, good/bad bed side manner – explicit mentions, can co-code with communication if also referenced, good 'approach' with patients, gets along well with patients, rapport
Care Planning	anticipates contingencies, quality care planning, forethought, identifies resources appropriately “thinking” skills, orders appropriate labs, initiates appropriately on work-ups/labs/images/etc, is thorough with their plan, managing skills, includes pt education
Clinical Skills	Comments on H&P , can co-code with assessment or procedural skill if mentioned

© 2022 Mamtani M et al. *JAMA Network Open.*

Assessments	Patient assessment
Procedural Skills	Any comment on any procedure
Decision Making	Well-reasoned, Good Judgement, evidence based, comments on decision making generally, MDM – medical decision making
Differential Diagnoses	Good differentials, considers all options, keeps open mind, doesn't 'close early'
Documentation	Writes thorough notes, needs to work on notes, sign-outs (code as both presentation and documentation unless specified)
Follow-Through	Follow-up on miscellaneous care plan action items like tasks, labs, work-ups, communication etc. timeline implied, executes appropriate
Knowledge Base	If individual needs to build their knowledge base or has a solid base
Patient Advocacy	patient supporter, goes to bat, pays overt attn. to patients/family members, awareness of psych/social context
Patient Presentation	oral presentation to other clinicians, case presentation
Preparedness for Independent Practice	Readiness of individual for independent practice – explicit to 'ready for independent practice', will do well in independent practice, or not ready for

© 2022 Mamtani M et al. *JAMA Network Open.*

**Management Style**	
Communication - Patient	Communication with patient/family
Communication – Provider	Communication ability with other providers, including residents, nurses, attendings, etc.
Dept Level Management	Anticipates bottlenecks delays in care, Good prioritization skills, triages well, thinks about dept. flow/needs
Efficiency	Timely, prompt, quick, efficient
Leadership	Takes Charge, leads team, takes initiative, takes ownership, manages team
Multitasking	Ability to manage several things at once, task switching
Organization	Comments on individuals level of organization, or organization skills
Patient Load	Individual is managing an appropriate number of patients, individual needs to take on more patients
Teaching Skill	Good teacher, working with jr residents/students/etc
Team Orientation	Builds relationships, Helpful, supports team morale, cooperativeness, well-liked by staff
**Personality Traits**	

© 2022 Mamtani M et al. *JAMA Network Open.*

Attitude and Demeanor	Doesn't Complain/complains, positive attitude, good energy, smiling, personality
Confidence	Deliberate, Decisive, is gaining confidence, mature/maturity
Critical Thinking	Problem Solver, logical, chews on a problem, thoughtful
Diplomatic	weighs opposing views, remains objective, Open Minded
Independence	Autonomous, self-starter, growing regarding independence, needs to be more independent, manages issues independently
Intelligence	Intuitive, Bright, Smart, curious
Level of Compassion	Kind, caring, empathetic
Professionalism	Level of professionalism – is/is not professional appearing or acting
Receptiveness to feedback	Tries to Improve, Acknowledges and learns from errors, is not receptive or is combative to feedback, seeking feedback and opportunity to learn, eager to learn
Resiliency	Performs Under Pressure vs experiencing burn out, coping with stress of job/being a resident, stays composed on busy shifts
Trustworthiness	Trust, Reliable, Accountable, Responsible

© 2022 Mamtani M et al. *JAMA Network Open.*

Work Ethic	Works hard, diligent, tireless, motivated, self-motivated, good worker, committed, dedicated, conscientious, push to work hard
**Co-Codes**	
Above Level	Individual is performing “above level” or “above peers”, including references to PG1/2/3, can be in relation to peers or just expected level or “high level, best, etc” code full content unless distinct tone shift (this is great, but work on that)
At Level	Individual is performing “as expected” or “at appropriate level”
Below Level	Individual is performing “below level” or “below peers”
Feedback on Specific Case	Specific case or episode/event, patient focus
Improvements Made	Comments that state improvements resident has made over time, growth, progressing w/ optl specific area of improvement
No Comment	When no comment is left/made
Non-Specific Comment	Generic comment, along the lines of "keep up the hard work" "good job" - *limited to comments where this is the only statement.*

© 2022 Mamtani M et al. *JAMA Network Open.*

Suggestions for Improvement	Comments noting areas for improvement, ways in which the resident needs to grow.

© 2022 Mamtani M et al. *JAMA Network Open.*
